# Rupture of a Calyceal Diverticulum Secondary to Ureteroscopy: A Rare Complication

**DOI:** 10.1155/2018/9285671

**Published:** 2018-07-09

**Authors:** Tomoya Yamasaki, Takashi Yoshioka, Masaya Imoto, Hiroshi Aoki, Kei Fujio, Shinya Uehara, Hideo Otsuki

**Affiliations:** ^1^Department of Urology, Abiko Toho Hospital, 1851-1 Abiko, Abiko-shi, Chiba-ken 270-1166, Japan; ^2^Center for Innovative Research for Communities and Clinical Excellence (CiRC^2^LE), Fukushima Medical University, 1 Hikarigaoka, Fukushima-shi, Fukushima-ken 960-1295, Japan; ^3^Department of Urology, Kawasaki Medical School General Medical Center, Okayama, 2-6-1 Nakasange, Kita-ku, Okayama-shi, Okayama-ken 700-8505, Japan

## Abstract

We present a case of a 45-year-old female who experienced rupture of a right calyceal diverticulum caused by ureteroscopy. Fifteen hours after the operation, she had severe right flank pain and a high fever (38.9°C). Computed tomography revealed perinephric extravasation of urine and bleeding inside the diverticulum. We diagnosed rupture of a calyceal diverticulum; therefore, we continued antibiotic administration and pain relief medication. She became afebrile on postoperative day 4 and was discharged from the hospital on postoperative day 7. Owing to renal cortex thinning in the diverticula, ureteroscopy is associated with a risk of rupture of calyceal diverticula.

## 1. Introduction

Calyceal diverticula are congenital renal abnormalities, and stones are often found in calyceal diverticula [1, 2]. For stones secondary to calyceal diverticula, ureteroscopic lithotripsy (URSL) is often indicated because of its high success rates and low complication rates [3]. Although there are some reports of successful treatment of ureteral stones secondary to calyceal diverticula, there are few reports on complications caused by treatments. Herein, we report a rare case of rupture of a calyceal diverticulum caused by ureteroscopy.

## 2. Case Presentation

**Figure 1:**
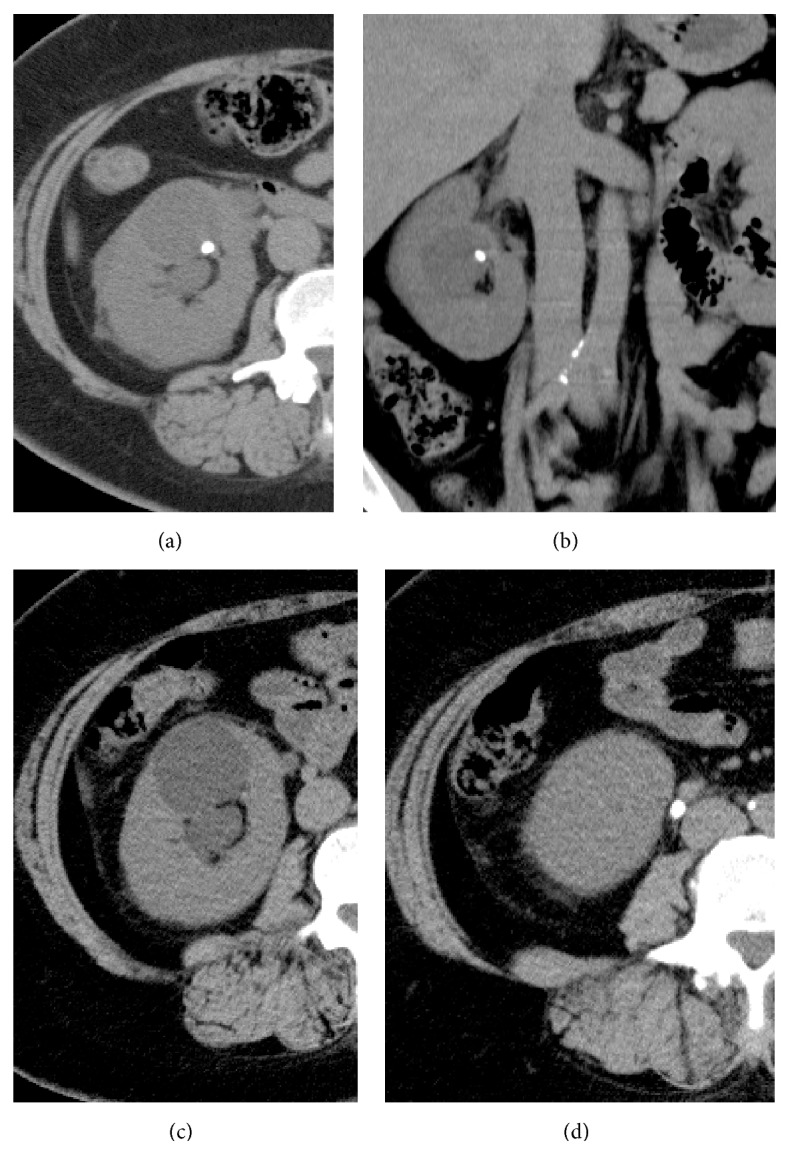
(a, b) Computed tomography image at the first visit showing a renal stone within a calyceal diverticulum of the right kidney. (c, d) The preoperative computed tomography image shows a right ureteral stone (5 × 5 mm) and hydronephrosis. The stone within the diverticulum seemed to have moved to the upper ureter.

A 45-year-old woman with severe right flank pain came to our outpatient clinic. Her past medical history included right ureteral calculi secondary to her right calyceal diverticulum. In addition to the past ureteral stone, there had also been a stone in the calyceal diverticulum. She had undergone URSL for a ureteral stone six months before this visit. Her past surgical history included a caesarian section. She was not taking any medication. The physical examination was within normal limits. Based on her past history, we suspected a recurrent ureteral stone. We performed computed tomography and diagnosed a right renal stone in her ureteropelvic junction (Figure 1). Her colic was very strong and she was very obese (her height was 162.8 cm, body weight was 97.6 kg, and body mass index was 36.8 kg/m^2^). Therefore, we decided that URSL was preferable, which was the same treatment as that used for the previous right ureteral stone.

Her preoperative urinalysis did not demonstrate bacteriuria, and her hematological exam and laboratory exam were within normal limits.

Ten days after the visit to our outpatient clinic, we performed URSL. Intravenous administration of ceftriaxone 1 g was started 30 min before ureteroscopy. The patient was placed in the lithotomy position and draped in a sterile fashion, under general anesthesia. First, the urethra and the bladder were observed, and the bilateral ureteral orifices were identified using a 22.5 Fr rigid urethrocystoscope (Cystoscopes, Olympus, Tokyo, Japan). Second, a semirigid 6/7.5 Fr ureteroscope (Ultrathin, Richard Wolf, Knittlingen, Germany) was inserted into the right ureter without a guidewire. Observation of the ureter was performed, but there were no ureteral stones in the ureteropelvic junction. We inserted a guidewire, and 12 Fr digital flexible ureteroscope (URF-V, Olympus) was moved into the right renal pelvis. Intermittent irrigation was controlled manually at the lowest pressure with a 50-ml syringe. We observed all calyces systematically in order to confirm the targeted stone. However, we were unable to identify the ureteral stone or the calyceal diverticulum. Finally, we stopped the operative procedure. The operative duration was 38 minutes, and the ureteroscopic duration was 25 minutes. Because of the short duration of the operation, we did not place a ureteral stent.

**Figure 2:**
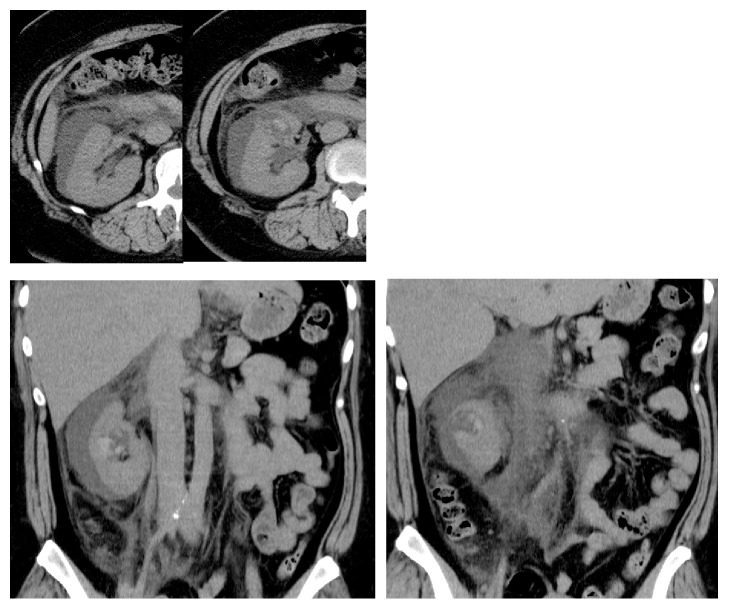
Computed tomography image on postoperative day 1 showing perinephric extravasation of urine and collapse of calyceal diverticulum. A high-density area of the diverticulum indicated bleeding due to rupture.

Fifteen hours later, the patient felt severe right flank pain and became febrile (38.9°C). Computed tomography showed perinephric extravasation of urine and bleeding inside the diverticulum (Figure 2). We diagnosed a rupture of the calyceal diverticulum secondary to ureteroscopy. We examined the urine culture and started ceftriaxone 2 g per day. On postoperative day 4, she became afebrile, and on postoperative day 7, we stopped administration of ceftriaxone and started oral antibiotics (tebipenem pivoxil 300 mg per day) guided by the reported urine culture (*E.coli *with extended spectrum beta lactamase). On postoperative day 14, she was asymptomatic; therefore, she stopped taking oral antibiotics. In postoperative month 3, we performed computed tomography and confirmed complete resolution of extravasation.

## 3. Discussion

The patient in this report experienced rupture of a right calyceal diverticulum secondary to ureteroscopy. Regrettably, we could find no renal or ureteral stones during the operation. We speculate that the targeted stone was spontaneously passed while the patient had waited for URSL.

Rupture of calyceal diverticula is a rare complication but an important consideration for both patients who have calyceal diverticula and urologists who perform ureteroscopic procedures.

Calyceal diverticula are rare congenital outpouchings of the renal calyx. The prevalence is reported to be 0.21 to 0.6% of intravenous urograms in both adults and children [4–6]. In calyceal diverticula, upper urinary tract calculi often occur. They were found to occur at a rate of 48.9% in upper poles, 29.7% in middle poles, and 21.4% in in lower poles, respectively [2]. Once stones become symptomatic, operative interventions are indicated [2]. For diverticular stones, URSL is a good surgical option because it has a greater efficacy than shock wave lithotripsy, as well as lower complication rates and lower discomfort levels than percutaneous nephrolithotomy or laparoscopic nephrolithotomy [2, 3].

In general, URSL has relatively few postoperative complications. A global study conducted by the Clinical Research Office of the Endourological Society showed that the overall postoperative complication rates of URSL were 3.5% [7]. Most of them were classified as Clavien Grade I or II, and a rupture of renal pelvis or renal cortex was not reported [7].

Renal calyceal rupture is also a rare occurrence. Gershman et al. reported a descriptive review that showed the causes of renal forniceal or calyceal rupture [8]. In that review, there were only four iatrogenic ruptures out of 108, and there were no cases secondary to ureteroscopic procedures [8]. From these findings, we believe this is the first case report that shows a rupture of calyceal diverticulum secondary to ureteroscopy.

Two clinically relevant findings were observed in this case. First, we should have performed a preoperative examination that confirmed the presence of a stone in order to prevent the patient from unnecessary ureteroscopic procedures. Second, as there is renal cortex thinning in the calyceal diverticulum, anatomical fragility can exist. Therefore, when performing URSL for stones with calyceal diverticula, perioperative decompression of intrapelvic pressure via a ureteral access sheath [9] or a postoperative ureteral stent [10] should be considered in order to prevent rupture of calyceal diverticula.

## 4. Conclusion

Calyceal diverticula are rare but are often associated with upper urinary tract stones. When ureteroscopic lithotripsy is considered as a treatment, we should take into account the risk of rupture of calyceal diverticula, and insertion of a ureteral access sheath and/or a postoperative double J stent should be considered.
